# A Band That Causes Leaky Valves: Severe Mitral Regurgitation due to Left Atrial Fibrous Band—A Case Report and Literature Review

**DOI:** 10.1155/2019/2458569

**Published:** 2019-03-19

**Authors:** Christopher Nnaoma, Gurdarshan Sandhu, Christoph Sossou, Ilan Vavilin, Jose Bustillo, Anshu Garg

**Affiliations:** ^1^Newark Beth Israel Medical Center, Newark, NJ, USA; ^2^St. George's University, Grenada

## Abstract

Left atrial fibrous band is a rare clinical and echocardiographic finding characterized by the presence of a fibrous band attached to the mitral valve. Diagnosis is accomplished with transesophageal echocardiography (TEE), live 3D imaging, or cardiac MRI. Most patients are asymptomatic and incidental findings. However, in rare cases, an atrial fibrous band can produce symptoms such as dyspnea on exertion, fatigue, and lightheadedness secondary to mitral regurgitation (MR) which can lead to heart failure if unattended to. More serious complications such as cardioembolic phenomenon can occur. We herein report a case of a 55-year-old male with hypertension who presented with dyspnea on exertion and chest pain. Transthoracic echocardiography (TTE) showed mitral valve prolapse with moderate to severe mitral regurgitation. TEE showed an atrial fibrous band. Given the patient's poor exercise tolerance, he was taken to surgery for a mitral annuloplasty.

## 1. Introduction

The first case of left atrial fibrous band was reported in 1897 by Rollestone [1]. Left atrial fibrous bands are rare. Autopsy data suggest the prevalence of left atrial bands to be approximately 2% [2]. Its association with MR is exceedingly unique [2]. The clinical importance of these bands in the left atrium is still not clearly defined and should not be confused with the left ventricular false tendon [3]. A better understanding of the natural history and clinical implications of this condition may better help us to diagnose and manage these patients. We present a case of severe symptomatic mitral regurgitation caused by a left atrial fibrous band. The patient had surgical resection of the band and symptoms improved significantly post procedure.

## 2. Methods

In addition to the presented case report, we reviewed the literature to compare the clinical presentation and management of patients with a left atrial fibrous band. The review considered papers and publications written in the English language only. Keywords used for electronic search were left atrial fibrous band and mitral regurgitation. Definitions used for case inclusion were (a) diagnosis of left atrial band either by TTE, TEE, cardiac MRI, live 3D imaging, (b) abnormalities produced by left atrial bands, and (c) age greater than 18 years. Autopsy-reported cases were not included and no studies were excluded based on the date of publication.

## 3. Case Presentation

**Figure 1:**
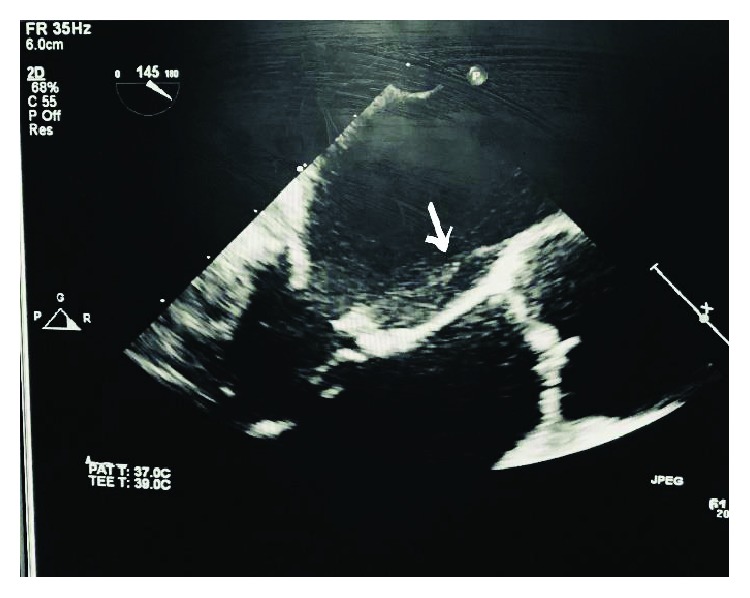
Preop ECHO showing mild focal bileaflet (anterior > posterior) leaflet prolapse with moderate to severe MR. EF was 45%.

**Figure 2:**
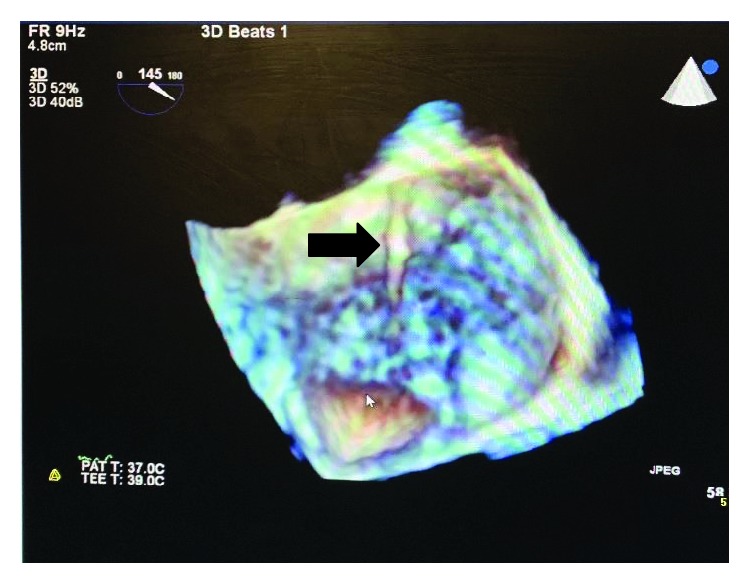
3D live imaging of the left atrial fibrous band.

A 55-year-old male with a past medical history of hypertension reports exertional dyspnea for 8 months and nonexertional chest pain for 5 days. Physical exam revealed an afebrile patient; BP of 133/82 mmHg; heart auscultation revealed a new 3/6 systolic murmur, maximal at the apex radiating to the axilla; no leg swelling; and no jugular vein distention. The lungs were clear to auscultation. Pertinent labs were a negative Troponin and BNP. CT angiography and cardiac catheterization were negative. Increased MR with exercise was noted. TTE revealed a moderate to severe posteriorly directed MR which was said to be functional in origin (Carpentier classification III). Ejection fraction (EF) was 45%. TEE showed a structure attached to the atrial surface of the anterior (A2, A3 component) leaflet of the mitral valve (Figure 1), most consistent with a left atrial fibrous band. The distal portion of the band was attached to the interatrial septum in close relation to the aortic valve. The band appeared to restrict the motion of the A2, A3 component with consequent MR, giving it a tented appearance, while 3D live imaging allowed for complete visualization of the band attaching to the mitral valve (Figure 2).

**Figure 3:**
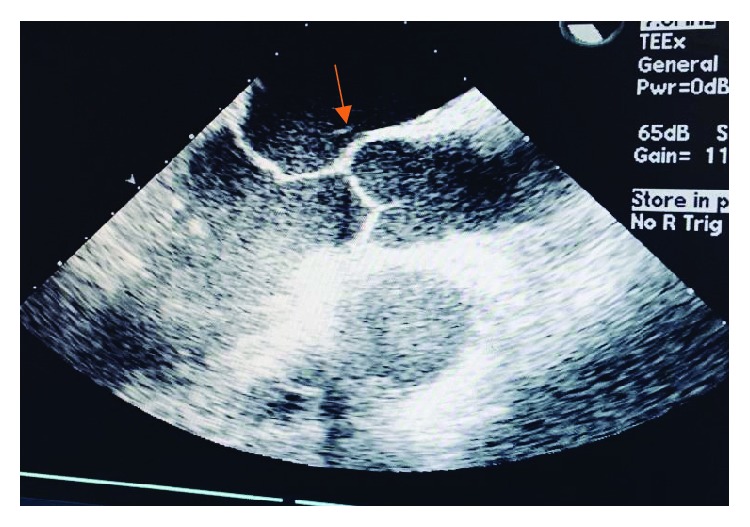
Intraoperative image of a thickened fibrous band from the atrial septum down to the edge of the A2 segment.

**Figure 4:**
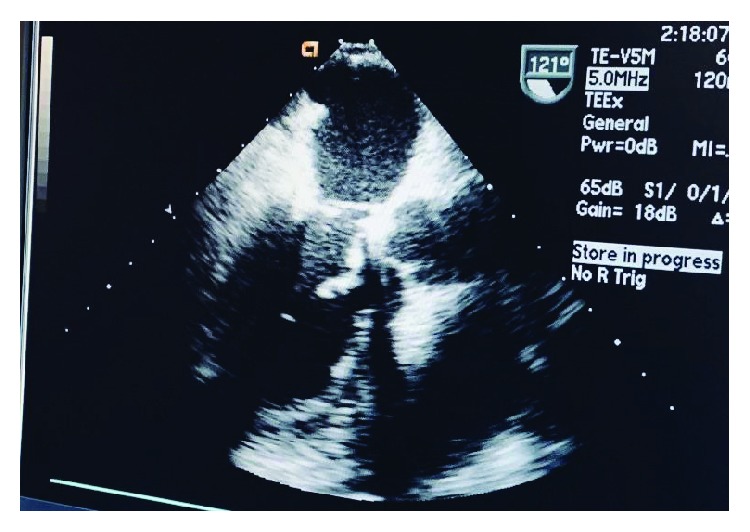
Postop ECHO showing mitral annulus after mitral annuloplasty with an EF of 49%.

Given his poor exercise tolerance, the patient was taken for mitral valve annuloplasty. Intraoperative findings included mitral valve prolapse (MVP), thickened fibrous mitral valve, and MR (Figure 3). The pathology of the fibrous band showed a cardiac valve/vascular wall-like tissue with focal degenerative changes. Three months after the repair, patient's symptoms improved significantly with good exercise tolerance. There were no audible murmurs. ECHO showed no mitral regurgitation about 3 months after mitral annuloplasty, with an EF of 49% (Figure 4).

## 4. Literature Review

To the best of our knowledge, fourteen cases of left atrial fibrous band have been described in the literature that met our inclusion criteria. These do not include those found incidentally on autopsy.

Table 1 summarizes the baseline demographics and associated clinical significance of these bands. There were ten males, four females, and one of undetermined sex. The mean age was 52.

**Figure 5:**
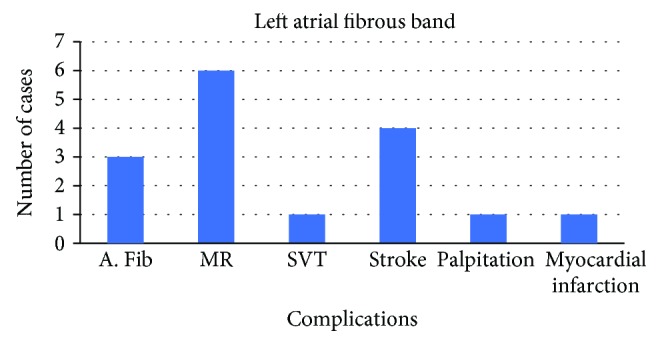
Incidence of complications in patients with atrial fibrous band.

Figure 5 represents a graphical presentation of these left atrial bands and their clinical significance.

The most common complication resulting from a left atrial fibrous band was MR which was present in about 37.5% of the cases. Most of the patients with MR progressed to congestive heart failure (CHF). 25% of cases were noted to have cryptogenic stroke and cardiac embolism. 18.7% of cases were noted to have atrial fibrillation (A. Fib) while 6.25% were from supraventricular tachycardia and myocardial infarction. The high incidence of stroke in these patients is likely due to increased incidence of patent foramen ovale (PFO) and A. Fib associated with left atrial fibrous band.

A graph showing the incidence of complications associated with left atrial fibrous bands is presented.

## 5. Discussion

Intracavitary muscle bands have been well described in all four chambers of the heart. It can be congenital, with no reported gender or racial predilection. Lev (1953) described three forms of abnormal partitioning of the left atrium which are distinguished by the location of the entrance of the venae cava and pulmonary veins [4]. The likely embryological explanation could be that the abnormal septum is likely to be part of the septum primum, which is deviated to the left side to form an extra subdivision of the left atrium [4].

Left atrial fibrous bands are said to be a very rare cause of MR. Yamashita et al. reported 22 cases of left atrial fibrous bands from 1,100 patient autopsies [5]. The mechanism of MR associated with left atrial fibrous bands can be due to prolapse (Carpentier type II) or restricted movement (Carpentier type IIIa or b). The incidence of Chiari's network was 27% in patients with left atrial fibrous band vs. 8% in people without (*P* < 0.01) while 23% had PFO as compared to 8% in people without left atrial fibrous band (*P* < 0.05). The increased amount of PFO in this group of patients predisposes them to an increased incidence of cardioembolic phenomenon [5]. Also, about 18% of the patients with left atrial fibrous band had A. Fib; this also predisposes them to increased risk of strokes.

There have also been four reported cases of cardioembolic events from left atrial bands [6], making up about 25% of cases reported. Most of these bands are either fibrous or fibromuscular. In our patient, the fibrous band may have originated from the atrial septum. These bands are usually congenital and can be found incidentally. The clinical significance of these bands is uncertain [7]; they are mostly asymptomatic. They are however relevant since they can be associated with mitral regurgitation [3, 8], valve cardiomyopathies, atrial fibrillation, conduction disorders, and infectious endocarditis and can interfere with the manipulation of the catheter during invasive procedures [9]. As noted above, these bands are mostly associated with mitral regurgitation, which can lead to heart failure if not treated early.

Definite diagnosis of these bands can be done through cardiac imaging modality (transthoracic echocardiography, transesophageal echocardiography, cardiac MRI, and 3D live imaging which allow for complete visualization of the band attaching to the mitral valve).

In our case, the band was not an incidental finding; the fibrous band likely originated from the atrial septum and produced significant symptom requiring surgical treatment which is the definite treatment. No PFO was noted in our patient.

## 6. Conclusion

Fibrous bands in the left atrium are rare and extremely rare causes of MR. When they do produce symptoms, they either can be managed medically or surgically depending on the symptoms and severity of MR. Surgery should be deferred in patients who are asymptomatic. Such patients should be followed up closely with scheduled echocardiography. We recommend that PFO be ruled out in these patients given a high associated incidence rate. Anticoagulation should be considered in those with A. Fib and PFO due to increased risk of cardioembolic strokes in these patients until a definite treatment is carried out.

## Figures and Tables

**Table 1:** Basic demographics and the clinical presentation of these patients.

Study	Age and sex	Complications
Irwin et al., Shin et al.	75-year-old male	Mitral regurgitation
Won Young Jang et al.	76-year-old male	Persistent atrial fibrillation
Olsen et al.	54-year-old male	Acute myocardial infarction
Saade et al.	49-year-old female	MR and palpitations
Sherif et al.	72-year-old male	MR leading to CHF
Bergaglio et al.	30-year-old male	SVT
Bergaglio et al.	67-year-old female	Ischemic right hemispheric stroke
Shunsuke Uetake et al.	72-year-old male	Paroxysmal atrial fibrillation
Ozer et al.	34-year-old male and 56-year-old female	Cryptogenic stroke
James Ker et al.	29-year-old male and 43-year-old male	Cardioembolic events leading to TIA
Okajima K. et al.	65-year-old male	Persistent AF
Barant T. et al.	Age and sex unknown	MR, MVP
Vlassak et al.	20-year-old female	Dyspnea and MR
Dawson D. et al.	33-year-old female	MR
